# Molecular characterization of *Trypanosoma cruzi* samples derived from *Triatoma vitticeps* and *Panstrongylus geniculatus* of the Atlantic rainforest, southeast Brazil

**DOI:** 10.1051/parasite/2018060

**Published:** 2018-11-26

**Authors:** Maria Augusta Dario, Tassiane Emanuelle Servare Andrade, Claudiney Biral dos Santos, Blima Fux, Adeilton Alves Brandão, Aloísio Falqueto

**Affiliations:** 1 Unidade de Medicina Tropical, Universidade Federal do Espírito Santo (UFES) Av. Marechal Campos, 1468 Vitória ES 29043-900 Brazil; 2 Núcleo de Entomologia e Malacologia, Secretaria de estado da Saúde (SESA/ES) Rua Pedro Zangradini, 320 Serra ES 29164-020 Brazil; 3 Laboratório Interdisciplinar em Pesquisas Médicas, Instituto Oswaldo Cruz/Fiocruz Av. Brasil, 4365 Rio de Janeiro RJ 21040-900 Brazil

**Keywords:** *Trypanosoma cruzi* discrete typing units, molecular epidemiology, Triatominae, Atlantic rainforest, Brazil

## Abstract

*Background*: In rural areas of Espírito Santo state, southeast Brazil, triatomine species attracted by light frequently invade residences. The aim of this study was to investigate the *Trypanosoma cruzi* discrete typing units (DTUs) harbored by these triatomines. *Methods*: Triatomine’s intestinal contents were examined, inoculated in mice, and the positive samples were cultivated. Flagellates obtained from infected mice hemoculture were submitted to DNA extraction using a salting-out method and to TcSC5D gene amplification. The amplified samples were sequenced, and polymorphism was analyzed for DTU identification. *Results*: Three hundred and ninety-four triatomines were identified: *Triatoma vitticeps* (90.03%), *Panstrongylus geniculatus* (8.89%), *Panstrongylus megistus* (0.54%), *Panstrongylus diasi* (0.27%), and *Triatoma tibiamaculata* (0.27%). Among the specimens, 251/394 (67.65%) presented flagellated forms similar to *T. cruzi*. After triatomine intestinal content inoculation into mice, 134 mice presented *T. cruzi*-like trypomastigotes from *Tr. vitticeps* and *P. geniculatus* and 89 samples were positive in hemoculture. Sixty-two samples were analyzed for the TcSC5D gene and TcI, TcII, TcIII, and TcIV DTUs were identified. *Conclusions*: We observed *T. cruzi* DTU diversity in *Tr. vitticeps* and *P. geniculatus*, which showed the predominance of TcII and occurrence of TcI, TcIII and TcIV. Triatomines presented high *T. cruzi* infection rates. Since little is known regarding the possible mammalian hosts that maintain the *T. cruzi* cycle, further studies are necessary to obtain a better understanding of the parasite transmission cycle in this region.

## Abstract (fr)

*Contexte* : Dans les zones rurales de l’État d’Espírito Santo, au sud-est du Brésil, des espèces de triatomines attirées par la lumière envahissent fréquemment les résidences. Le but de cette étude était d’étudier les unités discrètes de typage (DTU) de *Trypanosoma cruzi* hébergées par ces triatomines. *Méthodes* : Le contenu intestinal des triatomines a été examiné, inoculé à des souris et les échantillons positifs ont été cultivés. Les flagellés obtenus à partir d’hémocultures de souris infectées ont été soumis à une extraction d’ADN en utilisant une méthode de relargage et à une amplification du gène TcSC5D. Les échantillons amplifiés ont été séquencés et le polymorphisme a été analysé pour l’identification du DTU. *Résultats* : Trois cent quatre-vingt-quatorze triatomines ont été identifiées : *Triatoma vitticeps* (90.03 %), *Panstrongylus geniculatus* (8.89 %), *Panstrongylus megistus* (0.54 %), *Panstrongylus diasi* (0.27 %) et *Triatoma tibiamaculata* (0.27 %). Parmi les spécimens, 251/394 (67.65 %) présentaient des formes flagellées similaires à celles de *T. cruzi*. Après inoculation du contenu intestinal des triatomines à des souris, 134 souris ont présenté des trypomastigotes similaires à *T. cruzi* provenant de *Tr. vitticeps* et de *P. geniculatus* et 89 échantillons étaient positifs en hémoculture. Soixante-deux échantillons ont été analysés pour le gène TcSC5D et les DTU TcI, TcII, TcIII et TcIV ont été identifiés. *Conclusions* : Nous avons observé la diversité des DTU de *T. cruzi* chez *Tr. vitticeps* et *P. geniculatus*, qui a montré la prédominance de TcII et la présence de TcI, TcIII et TcIV. Les triatomines présentaient des taux d’infection élevés par *T. cruzi*. Comme on sait peu de choses sur les éventuels hôtes mammifères qui maintiennent le cycle de *T. cruzi*, des études supplémentaires sont nécessaires pour mieux comprendre le cycle de transmission du parasite dans cette région.

## Introduction

*Trypanosoma cruzi* is transmitted by blood sucking insects termed triatomines. Triatomines belong to the subfamily Triatominae, of which over 130 species are considered as potential *T. cruzi* vectors [21, 31]. In Brazil, 52 species and three genera: *Triatoma*, *Panstrongylus*, and *Rhodnius*, are registered as causing parasite transmission. Triatomine distribution is related to the overlap of their habitat with human colonization in areas with natural Brazilian Cerrado and Caatinga biomes, whereas Atlantic rainforest fragment areas generally do not present foci of triatomine domiciliation [20].

According to the Instituto de Pesquisas da Mata Atlântica [27], during the last century, the Espírito Santo state (ES) Atlantic rainforest fragment was devastated by wood exploitation and agricultural activities, with only 8.15% of the natural vegetal cover still remaining. Seven triatomine species are currently reported to occur in this region: *Cavernicola pilosa* Barber, 1937, *Rhodnius domesticus* Neiva & Pinto, 1923, *Panstrongylus diasi* Pinto & Lent, 1946, *Panstrongylus geniculatus* (Latreille, 1811), *Panstrongylus megistus* (Burmeister, 1835), *Triatoma tibiamaculata* (Pinto, 1926), and *Triatoma vitticeps* (Stal, 1859). Although these species present sylvatic habits [21, 56], adults are captured by human dwellers inside residences in rural areas, mostly from the ES state mountainous region [30], and *Tr. vitticeps* have been found colonizing human dwellings [57, 58]. In this context, *Tr. vitticeps* constitutes the main captured species, representing high *T. cruzi* natural infection rates [17, 56, 60].

Serological inquiry in ES state showed a low prevalence (0.019%) of Chagas disease (CD) [4, 61], consistent with the ineffectiveness of *Tr. vitticeps* to act as a vector through the contaminative route, as adult specimens eliminate their excreta late after their blood meal [57]. Nevertheless, as adult specimens are constantly invading residences, the potential contact with humans includes the risk of parasite transmission by other routes, such as oral transmission. No studies have yet been performed regarding the *T. cruzi* population circulating in triatomine species from ES state, reinforcing the idea that little information is available related to the *T. cruzi* populations in non-endemic areas [64]. Accordingly, the aim of this study was to investigate the *T. cruzi* populations that are present in the triatomine species that invaded residences in rural areas of ES state, which will provide a better understanding of the transmission dynamics of the parasite in the area.

## Materials and methods

### Study area

ES state is located along the southeastern coast of Brazil, encompassing 78 municipalities in a 46.095 km^2^ area. The municipalities are grouped into four macro regions (Metropolitana, Norte, Central, and South) and ten micro regions (Metropolitana, Central Serrana, Sudoeste Serrana, Litoral Sul, Central Sul, Caparaó, Rio Doce, Centro-Oeste, Nordeste, and Noroeste). The state covers the Atlantic rainforest central corridor, which comprises one of the main dense forest areas inside this biome [30].

### Triatomine capture

ES health agents were trained and instructed to visit rural area residents, teaching them to recognize, capture and preserve the triatomines that invaded their residences, as well as to handle the insects in an approved manner to avoid accidents. Upon capture of a triatomine by an individual in their residence, the insect was taken to a health center nearby or the municipality health agents were contacted to retrieve the triatomines from the residence.

The triatomine species from different localities within ES state collected by residents were delivered by municipality health agents to Núcleo de Entomologia e Malacologia from Secretaria de Estado da Saúde do Espírito Santo (NEMES – SESA/ES), located at Departamento de Patologia, Centro Ciências da Saúde, Universidade Federal do Espírito Santo (CCS/UFES), between June 2010 and May 2012. The collection of all the triatomines received was catalogued.

### Triatomine identification, examination, and intestinal content inoculation in mice

Triatomine identification was performed according to Lent and Wygodzinsky [31]. The insect digestive tract was dissected using forceps and scissors. The intestinal contents were diluted in saline solution (0.85%) and examined using an optical microscope to examine flagellate forms similar to *T. cruzi*. Upon positive results, the intestinal content was inoculated into Swiss albino mice by the intraperitoneal route. To confirm parasitemia by *T. cruzi*, the mice were examined every 15 days to observe the presence of trypomastigote bloodstream forms. The animals were examined up to the 60th day. In case of positivity in blood or negativity after the 60-day period, the animals were sacrificed using methods recommended by the Universidade Federal do Espírito Santo Animal Ethics Committee (pentobarbital sodium 40 mg/kg; intraperitoneal route).

### Hemoculture examination

Blood from positive mice was collected by cardiac puncture under anesthesia (acepromazine (2%) in 9:1 ketamine hydrochloride (10%)) for subsequent hemoculture in MacNeal, Novy, and Nicole (NNN) medium with Brain Heart Infusion (BHI) overlay, and incubated at 28 °C. The hemocultures were examined under an optical microscope every 15 days until the 60th day. Subsequently, if the hemocultures did not present *T. cruzi* epimastigote forms, they were discarded. Positive hemocultures were washed with 1 mL of phosphate buffer solution (pH 7.2) and centrifuged at 448 × *g* for 10 min. The supernatant was discarded, and the pellet was stored at −20 °C until DNA extraction.

### *Trypanosoma cruzi* molecular characterization

*Trypanosoma cruzi* DNA extraction was performed using a salting out method [2]. For *T. cruzi* genotyping, the DNA was submitted to polymerase chain reaction (PCR) for the TcSC5D marker using the following primers: TcSC5D-fwd 5′-GGACGTGGCGTTTGATTTAT-3′ and TcSC5D-rev 5′-TCCCATCTTCTTCGTTGACT-3′ [12], as previously described. In all PCR reactions, we used positive controls and water as a negative control, to validate the reaction.

The 832-bp fragment obtained was purified using a NucleoSpin Extract II purification kit (Macherey-Nagel, Düren, Germany) and then the products were sequenced for the determination of *T. cruzi* genotypes using a BigDye terminator v3.1 cycle sequencing kit (Applied Biosystems, Foster City, CA, USA) on an ABI 3730 DNA sequencer available at the PDTIS/FIOCRUZ sequencing facility. The sequences were edited and corrected using BioEdit software and compared with *T. cruzi* nucleotide sequences deposited in GenBank using the Basic Local Alignment Search Tool (BLAST) algorithm. The sequences obtained were aligned with reference strain sequences (TcI: X10 – JN050585, DM28 – JN050567; TcII: Y – JN050587, IVV – JN050569; TcIII: M6241 – JN050573, M5631 – JN050572; TcIV: CanIII – JN050566, 91122102R – JN050564; TcV: LL014 – JN050570, Sc43 – JN050578; and TcVI: ClBrener – XM797152, Tula – JN050584) and the polymorphisms associated with each genotype were noted, according to those described in Cosentino and Agüero [12], using MEGA5 software [66]. The sequences obtained were deposited in GenBank with accession numbers KY056665–KY056726.

### Ethics statement

This study was approved by the Ethical Animal Use Committee of the Federal University of Espírito Santo (CEUA/UFES), protocol number: 016/2010.

## Results

### Triatomine capture rates and natural *T. cruzi* infection

In our study, 394 triatomine specimens were captured in 25 ES municipalities between June 2010 and May 2012, of which 371 were adult specimens and 23 were nymphs. Most were captured in domestic environments, mainly in bedrooms. The adult specimens were identified as: *Triatoma vitticeps* (334; 90.03%), *Panstrongylus geniculatus* (33; 8.89%), *Panstrongylus megistus* (2; 0.54%), *Panstrongylus diasi* (1; 0.27%), and *Triatoma tibiamaculata* (1; 0.27%). All the captured nymphs were from *Tr. vitticeps* species. Among the 394 captured triatomines, 251 (67.65%) presented flagellated forms similar to *T. cruzi* (Table 1). None of the 23 nymphs presented such flagellated forms.

**Table 1:** Triatomine species and *Trypanosoma cruzi* infection rates.

Triatomine species	Specimens infected
*Triatoma vitticeps*	241 (96.02%)
*Panstrongylus geniculatus*	9 (3.59%)
*Panstrongylus megistus*	1 (0.39%)
Total	251 (100%)

### Hemoculture and *T. cruzi* genotyping

Following inoculation into mice of 251 triatomine intestinal contents, 134 mice presented with *T. cruzi* trypomastigote forms: 127 were from the intestinal contents of *Tr. vitticeps* and seven from *P. geniculatus*. Of the 134 hemocultures, 89 presented with *T. cruzi* epimastigote forms, of which 85 samples belonged to *Tr. vitticeps* and four belonged to *P. geniculatus*.

**Figure 1:**
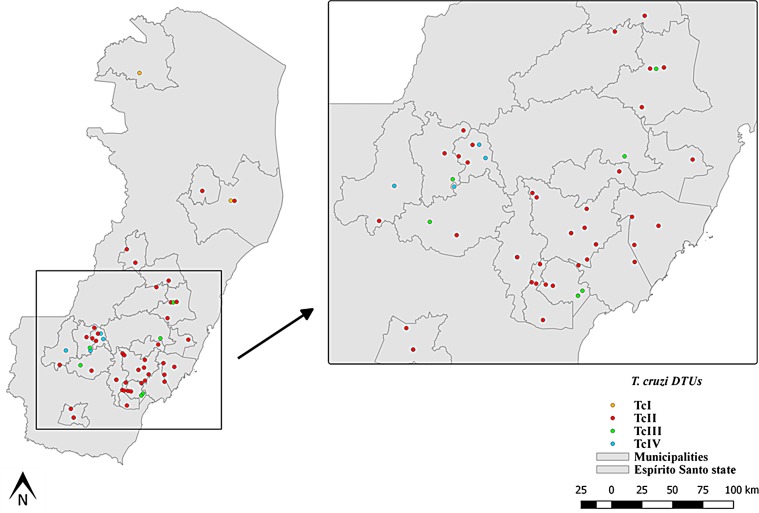
*Trypanosoma cruzi* DTU distribution in Espírito Santo state. DTUs are coded by color: orange, DTU TcI; red, DTU TcII; green, DTU TcIII; blue, DTU TcIV.

DNA extraction was performed for 89 cultures in which the presence of *T. cruzi* was evident by microscopy, and PCR amplification products for the TcSC5D gene were obtained for 62 samples. These were genotyped as representing four *T. cruzi* DTUs circulating in *Tr. vitticeps* (58 samples) and *P. geniculatus* (four samples). Of these, 50 samples (80.65%) were genotyped as DTU TcII, five (8.06%) as TcIII, five (8.06%) as TcIV, and two (3.22%) as TcI (Tables 2 and 3). This result indicates that in ES state, triatomines demonstrate high diversity of *T. cruzi* DTUs circulating in the sylvatic environment (Fig. 1).

**Table 2:** *Trypanosoma cruzi* DTU identification by triatomine species.

Triatomine species	*Trypanosoma cruzi* DTU			
	TcI	TcII	TcIII	TcIV
*Triatoma vitticeps*	2	48	3	5
*Panstrongylus geniculatus*	–	2	2	–
Total	2	50	5	5

**Table 3:** Alignment sequences from *Trypanosoma cruzi* TcSC5D isolated from *Triatoma vitticeps* and *Panstrongylus geniculatus*.

*T. cruzi* strain/sample	Nucleotide position							
	92	131	290	356	383	449	611	620
Dm28c (TcI)	A	T	C	T	T	T	T	C
X10 (TcI)	.	G	.	.	.	.	.	.
67	.	G	.	.	.	.	.	.
115	.	.	.	.	.	.	.	.
Y (TcII)	T	C	T	A	C	.	G	A
IVV (TcII)	T	C	T	A	C	.	G	A
19	T	C	T	A	C	.	G	A
030	T	C	T	A	C	.	G	A
075	T	C	T	A	C	.	G	A
160	T	C	T	A	C	.	G	A
235	T	C	T	A	C	.	G	A
134	T	C	T	A	C	.	G	A
001	T	C	T	A	C	.	G	A
21	T	C	T	A	C	.	G	A
031	T	C	T	A	C	.	G	A
055	T	C	T	A	C	.	G	A
083	T	C	T	A	C	.	G	A
165	T	C	T	A	C	.	G	A
214	T	C	T	A	C	.	G	A
241	T	C	T	A	C	.	G	A
021	T	C	T	A	C	.	G	A
032	T	C	T	A	C	.	G	A
086	T	C	T	A	C	.	G	A
138	T	C	T	A	C	.	G	A
168	T	C	T	A	C	.	G	A
003	T	C	T	A	C	.	G	A
23	T	C	T	A	C	.	G	A
034	T	C	T	A	C	.	G	A
109	T	C	T	A	C	.	G	A
196	T	C	T	A	C	.	G	A
217	T	C	T	A	C	.	G	A
209	T	C	T	A	C	.	G	A
023	T	C	T	A	C	.	G	A
041	T	C	T	A	C	.	G	A
113	T	C	T	A	C	.	G	A
152	T	C	T	A	C	.	G	A
201	T	C	T	A	C	.	G	A
010	T	C	T	A	C	.	G	A
024	T	C	T	A	C	.	G	A
045	T	C	T	A	C	.	G	A
153	T	C	T	A	C	.	G	A
203	T	C	T	A	C	.	G	A
221	T	C	T	A	C	.	G	A
11	T	C	T	A	C	.	G	A
025	T	C	T	A	C	.	G	A
127-E	T	C	T	A	C	.	G	A
156	T	C	T	A	C	.	G	A
016	T	C	T	A	C	.	G	A
29	T	C	T	A	C	.	G	A
052	T	C	T	A	C	.	G	A
073	T	C	T	A	C	.	G	A
129	T	C	T	A	C	.	G	A
132	T	C	T	A	C	.	G	A
159	T	C	T	A	C	.	G	A
231	T	C	T	A	C	.	G	A
205	T	C	T	A	C	.	G	A
M6241 (TcIII)	G	.	.	G	.	G	C	.
M5631 (TcIII)	G	.	.	A	.	G	C	.
143	G	.	.	G	.	G	C	.
054	G	.	.	G	.	G	C	.
049	G	.	.	A	.	G	C	.
229	G	.	.	A	.	G	C	.
065	G	.	.	G	.	G	C	.
210	G	.	.	A	.	G	C	.
CanIII (TcIV)	T	.	A	A	.	.	A	A
921221 (TcIV)	T	.	A	A	.	.	A	A
066	T	.	A	A	.	.	A	A
064	T	.	A	A	.	.	A	A
215	T	.	A	A	.	.	A	A
062	T	.	A	A	.	.	A	A
LL014 (TcV)	K	Y	Y	R	Y	K	S	M
Sc43 (TcV)	K	Y	Y	A	Y	K	S	M
CL Brener (TcVI)	T	C	T	A	C	.	G	A
Tula (TcVI)	K	C	Y	R	Y	K	S	M
*T. c. marinkellei*	T	C	T	A	T	A	A	A

The dots represent the same base position. *Trypanosoma cruzi marinkellei* (KC881179) was used as the outgroup.

## Discussion

Although only three cases of autochthonous CD have been reported in ES state since 2007, according to the Espírito Santo State Health Department (SESA/ES), triatomines frequently invade residences in rural areas of ES state resulting in the dwelling inhabitants being in contact with this parasite. However, only studies addressing *T. cruzi* infection in triatomines have been mostly reported to date. In contrast, the current report comprises a recent study demonstrating that together with the high rates of *T. cruzi* infection, triatomines represent a source of considerable *T. cruzi* genotype diversity circulating in a given biome.

In this study, we identified five different triatomine species that occur in ES state: *Tr. vitticeps*, *P. geniculatus*, *P. megistus*, *P. diasi*, and *Tr. tibiamaculata*, representing the same species that were found by Leite et al. [30] during an earlier study regarding triatomines in ES state. The occurrence of *C. pilosa* and *R. domesticus* species has been described previously [21, 25], but these species were not identified in the current study. Although *Tr. vitticeps* is considered a sylvatic species, it is a secondary vector of CD transmission by the vectorial contaminative route, and has been found frequently in the intradomiciliar environment. This triatomine species exhibits a high *T. cruzi* infection rate, which has previously been reported in ES as well as in Rio de Janeiro and Minas Gerais states [22, 55, 58, 59]. In the present study, among the 67.65% of triatomines infected with *T. cruzi*, 64.96% were from *Tr. vitticeps*. This high *T. cruzi* infection rate demonstrates a relationship between this triatomine and sylvatic mammals, indicating the capacity of this species to maintain the sylvatic cycle in ES state [57, 58].

*Panstrongylus geniculatus* and *P. megistus* were also found to be infected with *T. cruzi*, although with a *T. cruzi* infection rate lower than that of *Tr. vitticeps*. *Panstrongylus geniculatus* is widely distributed in Latin America [29]. This species is correlated with the enzootic cycle and the transmission of CD in Venezuela and Colombia [9, 11, 18, 23]. In Brazil, the species is found in Bahia and in the Amazon region [19, 51, 54, 67, 68]. Notably, natural *T. cruzi* infection by *P. geniculatus* has previously been identified only in the Amazon region [51]; the current finding of 33 *P. geniculatus* specimens from ES state is the first report in the Atlantic rainforest region. *Panstrongylus megistus* is one of the most important triatomine species domiciliated in Cerrado and Caatinga biomes and can be found from northeast to south Brazil as well as in Bolivia, Paraguay, Uruguay, and Argentina [47, 63]. A study performed by Paula et al. [48] in Minas Gerais state demonstrated that *P. megistus* exhibited a *T. cruzi* infection rate of 8.3% and that from among a total of 147 specimens identified in Brasilia Federal District, only three (1.45%) presented flagellate forms [36]. These results reinforce the low *T. cruzi* infection rate exhibited by *P. megistus*, which can be justified by its correlation with chicken houses in the peridomicile [5, 48, 69]. However, although *P. megistus* has been shown to represents an effective CD vector and to be present in domiciles in Brazilian Cerrado and Caatinga biomes, in the Atlantic rainforest it is predominantly sylvatic, as was also observed by Barbosa et al. [3] in Santa Catarina state.

*Trypanosoma cruzi* is a genetically diverse parasite: six *T. cruzi* populations are recognized, denoted as TcI to TcVI, as well as a seventh DTU termed TcBat [38, 71, 72]. Notably, in Brazil, the existence of a heterogeneous *T. cruzi* population supporting the occurrence of the six *T. cruzi* DTUs has been reported [6, 14, 43, 65]. In the present study, the *T. cruzi* genotypes circulating in triatomines from the Atlantic rainforest were found to be genetically heterogeneous, with the predominance of DTU TcII and DTUs TcI, TcIII, and TcIV were also presented. This DTU diversity was observed in Guarapari municipality, ES state, in which the DTUs TcI, TcII, TcIII and TcIV were identified infecting triatomines [15, 16].

DTU TcII is found in the southern cone of South America [72], although its occurrence has been reported in the Amazon region, Colombia, Mexico, and the United States [26, 32, 37, 50]. According to Miles et al. [44], TcII is rarely found in the sylvatic cycle; however, in the Atlantic rainforest, it has been reported in primates and has been isolated from the armadillo (*Euphractus sexcinctus*) as well [33, 34, 70]. Notably, whereas all CD vectors in ES state are in sylvatic areas, the occurrence of DTU TcII was predominant in this environment. As *T. cruzi* is known to circulate in more than 100 mammal species [28], this genotype likely already existed in this environment prior to triatomine domiciliation. DTU TcI has a wide distribution on the American continent and is responsible for CD cases in Central and Northern South America. It is correlated to the arboreal transmission cycle in nature and opossums from *Didelphis* sp. comprise the main reservoir [9, 72, 42, 7, 68, 24]. Although TcI is very commonly found in nature, in ES state only two samples were identified in *Tr. vitticeps*, which contrasts with a previous report of infection of this triatomine by DTU TcI at a high percentage [53].

In turn, DTU TcIII can be found in different regions in Brazil; this genotype has been found to be responsible for cases of CD in North, Northeast, Southeast, and South Brazilian regions [1, 35, 40, 45]. It is correlated to the terrestrial transmission cycle, with its main vector being *P. geniculatus* species [39]. In the present study, we described the occurrence of DTU TcIII in *P. geniculatus* and in *Tr. vitticeps*. In comparison, according to Cardinal et al. [8] and Martins et al. [41], TcIII was described as infecting triatomines from *T. rubrovaria* and *T. infestans*, respectively. The demonstration of the infection of additional triatomine species by this genotype reinforces the notion that the correlations between single species and genotypes remain poorly understood. Finally, DTU TcIV is encountered from the southern United States to northern South America and, together with TcI, is responsible for CD cases in the Amazon region [10, 24, 46, 50, 52]. The reporting of TcIV in *Tr. vitticeps* in ES state shows that its distribution is more extensive than has been reported. This finding also reinforces the Amazon enclave theory proposed by Costa [13], which states that a thousand years ago, the Atlantic rainforest and the Amazon were connected. In support of this theory, Pinto et al. [49] and Silva et al. [62] reported the appearance of distinct species of sandflies and *Anopheles* mosquitoes from the Amazon in the ES Atlantic rainforest. Our report of *T. cruzi* populations that occurred both in the Amazon and in the Atlantic rainforest thus serves to further support this theory.

This study is the first report of *T. cruzi* DTUs in different municipalities from ES state, as prior studies were limited to investigating only *T. cruzi* infection rates in triatomines. Our results demonstrate that *T. cruzi* DTU distribution is much broader than has previously been reported; for example, TcII, which was associated with the domestic transmission cycle, was found to be widely distributed in nature. Furthermore, it was observed that the small Atlantic rainforest fragment studied herein presented marked diversity of circulating DTUs and that one triatomine species, *Tr. vitticeps*, hosted four different *T. cruzi* genotypes. For future studies, it is necessary to understand the ecological scenario that maintains DTU diversity in this biome.

## Conflict of interest

The authors declare that they have no competing interests.
